# Supplementary material for: Development of a computer-aided design software for the quantitative evaluation of aesthetic damage
Source: PLoS One. 2019 Dec 18;14(12):e0226322. doi: 10.1371/journal.pone.0226322 (PMC6919621; doi:10.1371/journal.pone.0226322)
Supplement: S1 Code site — 1. (ZIP) [file pone.0226322.s001.zip › [EESC_jr][Projeto]Relatorio_07_21/vendors/select2/docs/_includes/options/data/array.html]

## Can I load data into Select2 using an array?

While Select2 is designed to be used with a `<select>` tag
the data that is used to search through and display the results can be
loaded from a JavaScript array using the `data` option. This
option should be passed in during the initialization of Select2.

{% highlight js linenos %}
$('select').select2({
data: [
{
id: 'value',
text: 'Text to display'
},
// ... more data objects ...
]
});
{% endhighlight %}

### What properties are required on the objects passed in to the array?

The `id` and `text` properties are required on each
object, and these are the properties that Select2 uses for the internal
data objects. Any additional paramters passed in with data objects will be
included on the data objects that Select2 exposes.

### Do the `id` properties have to be strings?

Because the `value` attributes on a `>select<`
tag must be strings, the `id` property on the data objects must
also be strings. Select2 will attempt to convert anything that is not a
string to a string, which will work for most situations, but it is
recommended to force all of your ids to strings ahead of time.

### I can't select results with blank ids or an id of `0`!

See Do the `id` properties have to be strings?.

### How should nested results be formatted?

Nested results should be specified using the `children` property
on the data objects that are passed in. This `children` property
should be an array of data objects that are grouped under this option, and
the label for the group should be specified as the `text`
property on the root data object.

{% highlight js linenos %}
{
text: 'Group label',
children: [
{
id: 'nested-1',
text: 'First nested option'
},
// ... more data objects ...
]
}
{% endhighlight %}

### How many levels of nesting are allowed?

Because Select2 falls back to an `<optgroup>` when
creating nested options, only
a single level of nesting
is supported. Any additional levels of nesting is not guarenteed to be
displayed properly across all browsers and devices.

### Why are `<option>` tags being created?

The `data` option is a shortcut that Select2 provides which
allows you to load options into your `select` from a data array.

{% include options/not-written.html %}

### My objects don't use `id` for their unique identifiers, what can I do?

Select2 requires that the `id` property is used to uniquely
identify the options that are displayed in the results list. If you use a
property other than `id` (like `pk`) to uniquely
identify an option, you need to map your old property to `id`
before passing it to Select2.

If you cannot do this on your server or you are in a situation where the
identifier cannot be changed, you can do this in JavaScript before passing
it to Select2.

{% highlight js linenos %}
var data = $.map(yourArrayData, function (obj) {
obj.id = obj.id || obj.pk; // replace pk with your identifier
return obj;
});
{% endhighlight %}

### My objects use a property other than `text` for the text that needs to be displayed

Just like with the `id` property, Select2 requires that the text
that should be displayed for an option is stored in the `text`
property. You can map this property from any existing property using the
following JavaScript.

{% highlight js linenos %}
var data = $.map(yourArrayData, function (obj) {
obj.text = obj.text || obj.name; // replace name with the property used for the text
return obj;
});
{% endhighlight %}
